# The association between organophosphate insecticides and blood pressure dysregulation: NHANES 2013–2014

**DOI:** 10.1186/s12940-022-00887-3

**Published:** 2022-08-08

**Authors:** Frank Glover, Michael L. Eisenberg, Federico Belladelli, Francesco Del Giudice, Tony Chen, Evan Mulloy, W. Michael Caudle

**Affiliations:** 1 Gangarosa Department of Environmental Health, Rollins School of Public Health, Emory University, Atlanta, GA 30322 USA; 2 Department of Maternal-Infant and Urological Sciences, “Sapienza” Rome University, Policlinico Umberto I Hospital, Rome, Italy; 3 Department of Urology, Stanford University School of Medicine, Stanford, CA 94305 USA

**Keywords:** Hypertension, Blood pressure, Insecticides, Endocrine disruption, Risk assessment

## Abstract

**Background:**

Organophosphate (OP) insecticides represent one of the largest classes of sprayed insecticides in the U.S., and their use has been associated with various adverse health outcomes, including disorders of blood pressure regulation such as hypertension (HTN).

**Methods:**

In a study of 935 adults from the NHANES 2013–2014 cycle, we examined the relationship between systolic and diastolic blood pressure changes and urinary concentrations of three OP insecticides metabolites, including 3,5,6-trichloro-2-pyridinol (TCPy), oxypyrimidine, and *para*-nitrophenol. These metabolites correspond to the parent compounds chlorpyrifos, diazinon, and methyl parathion, respectively. Weighted, multivariable linear regression analysis while adjusting for potential confounders were used to model the relationship between OP metabolites and blood pressure. Weighted, multivariable logistic regression analysis was used to model the odds of HTN for quartile of metabolites.

**Results:**

We observed significant, inverse association between TCPy on systolic blood pressure (β-estimate = -0.16, *p* < 0.001) and diastolic blood pressure (β-estimate = -0.15, *p* < 0.001). Analysis with *para*-nitrophenol revealed a significant, positive association with systolic blood pressure (β-estimate = 0.03, *p* = 0.02), and an inverse association with diastolic blood pressure (β-estimate = -0.09, *p* < 0.001). For oxypyrimidine, we observed significant, positive associations between systolic blood pressure (β-estimate = 0.58, *p* = 0.03) and diastolic blood pressure (β-estimate = 0.31, *p* < 0.001). Furthermore, we observed significant interactions between TCPy and ethnicity on systolic blood pressure (β-estimate = 1.46, *p* = 0.0036). Significant interaction terms were observed between oxypyrimidine and ethnicity (β-estimate = -1.73, *p* < 0.001), as well as oxypyrimidine and BMI (β-estimate = 1.51 *p* < 0.001) on systolic blood pressure, and between oxypyrimidine and age (β-estimate = 1.96, *p* = 0.02), race (β-estimate = -3.81 *p* = 0.004), and BMI on diastolic blood pressure (β-estimate = 0.72, *p* = 0.02). A significant interaction was observed between *para-*nitrophenol and BMI for systolic blood pressure (β-estimate = 0.43, *p* = 0.01), and between *para*-nitrophenol and ethnicity on diastolic blood pressure (β-estimate = 2.19, *p* = 0.006). Lastly, we observed a significant association between the odds of HTN and TCPy quartiles (OR = 0.65, 95% CI [0.43,0.99]).

**Conclusion:**

Our findings support previous studies suggesting a role for organophosphate insecticides in the etiology of blood pressure dysregulation and HTN. Future studies are warranted to corroborate these findings, evaluate dose–response relationships between organophosphate insecticides and blood pressure, determine clinical significance, and elucidate biological mechanisms underlying this association.

**Supplementary Information:**

The online version contains supplementary material available at 10.1186/s12940-022-00887-3.

## Introduction

Hypertension (HTN) poses a significant public health and economic burden, and it is estimated that over 100 million U.S. adults are currently living with HTN [1–3]. Hypertension is estimated to cost over $130 billion annually, and is the leading cause of morbidity and mortality associated with cardiovascular diseases and strokes [4]. Clinically, HTN can be defined as a systolic blood pressure of 140 mmHg or greater, and/or a diastolic blood pressure of 90 mmHg or greater [5]. Recently, studies have shown that even modest deviations from normal blood pressure can significantly increase ones chances of adverse cardiovascular events, and patients with a systolic blood pressure between 120–139 mmHg and/or diastolic pressure between 80–89 are considered pre-hypertensive [6]. In over 80% of cases, the exact cause of HTN is unknown, and these situations are classified as primary or “essential” HTN [5]. In contrast, secondary HTN describes a situation where a known cause for the pathology has been determined (e.g. side effect of specific medications, genetic conditions such as hyperaldosteronism, organ dysfunction, etc.) [3, 7]. While lifestyle factors, diet, aging, and genetic predispositions have been strongly linked with the occurrence of HTN, the influence of exposure to environment chemicals on the initiation and/or progression of HTN has recently gained more attention [8–12].

Historically, a variety of toxicants have been associated with HTN in epidemiological and laboratory studies. Many of these chemicals are classified as persistent organic pollutants (POPs), and include compounds such as dioxin-like and non dioxin-like polychlorinated biphenyls (PCBs), phthalates, perfluorooctanoic acids (PFOAs), and various organochlorine insecticides like Dichlorodiphenyltrichloroethane (DDT) [13, 14]. While some of these chemicals have been phased out over time and their use restricted, the biochemical properties of POPs including lipophilicity and resistance to biodegradation increase their half-lives in the environment and biological compartments, and thus even restricted or banned chemicals can still contribute to adverse health effects in various populations years later [15]. Additionally, newer alternatives that share similar chemical properties have been shown to have similar deleterious effects on organ systems and overall health, most notably organophosphate insecticides.

In the U.S., organophosphate (OP) insecticides have been manufactured for decades, and millions of kilograms of these insecticides are produced and sprayed annually [16]. Currently, OP insecticides constitute roughly one-third of all insecticides used in the U.S., with the most common OP insecticide being chlorpyrifos [17]. While OP insecticides provided many benefits in crop yield and reduction in vector-borne illnesses, their strong associations with cholinergic toxicity and cognitive impairment in children following in utero exposures have raised public health concerns, resulting in their restricted use in many countries [18–20]. While parathion has been banned from both residential and agricultural use in the U.S. since 2000, methyl parathion, diazinon, chlorpyrifos, and methyl chlorpyrifos are still registered for agricultural use. As a result, metabolites of these insecticides are still readily quantifiable in the general population which reflects significant environmental exposures. The longevity of these insecticides is due in part to their chemical properties that make them highly lipid soluble, and resistant to biodegradation in certain environments [21]. Exposure to OP insecticides can occur via multiple pathways, including household and agricultural use, dietary exposure to insecticide residues, and exposure to agricultural drift [22]. Dietary exposure comes primarily from residues in fruits and vegetables, as well as contaminated meat, fish, rice, and dairy products. In one study, quantified levels of chlorpyrifos in commonly sold vegetables ranged from 0.01–3.5 mg/kg [23]. Public health initiatives and studies monitor OP metabolites in human samples such as urine, because these concentrations can serve as a reliable proxy for exposure to parent compounds like chlorpyrifos [24]. Several studies have shown that over 90% of the U.S. adult population has measurable levels of a specific metabolite of chlorpyrifos, TCPy, in their urine [25, 26]. Additionally, quantification of metabolite concentrations in the general population can give insights into the daily intake of parent compounds reaching systemic circulation. With this information, scientists can model the dose–response relationship between various concentrations of insecticides and health outcomes, and these results help public health experts and regulatory agencies like the Environmental Protection Agency (EPA) set cutoffs and guidelines defining safe doses, as well as providing evidence supporting restrictions on harmful chemicals.

Recent studies have begun investigating the relationship between OP insecticides and the risk for HTN. The primary mechanism of OP insecticides is inhibition of acetylcholinesterase, the enzyme responsible for breaking down acetylcholine [27, 28]. With this enzyme inhibited, a robust activation of acetylcholine-dependent (cholinergic) pathways ensues, resulting in overstimulation of cholinergic pathways. Many cholinergic pathways are involved in the central (brain) and peripheral (heart, kidneys, endothelium) control of vascular tone and heart rate, through connections with the sympathetic and parasympathetic nervous systems [29–32]. Perturbation of cholinergic pathways through inhibition of acetylcholinesterase has been hypothesized to be one way in which OP insecticides contribute to the pathogenesis of HTN. Chlorpyrifos, the most commonly used OP pesticide in the U.S., and diazinon have been associated with increased risk of gestational HTN in a cohort of migrant farmworkers, as well as elevations in blood pressure of children exposure to these chemicals during high-spray seasons [33–35]. A recent study by Javeres et. al found that chronic exposure to OP insecticides increases risk for metabolic disorders and HTN [10]. Additionally, subacute chlorpyrifos exposure in Wistar rats resulted in prolonged HTN and cardiometabolic abnormalities and a prior NHANES study found positive associations between non-specific metabolites of OP pesticides and adverse cardiometabolic health risk [36, 37]. In this cross-sectional study, we expand on the current literature to investigate the association between three specific metabolites of OP pesticides and blood pressure.

## Research design/methods

### National health and nutrition examination survey (NHANES)

Data analyzed was collected from the NHANES 2013–2014 survey cycle (available from:https://wwwn.cdc.gov/Nchs/Nhanes/2013-2014/TST_H.htm). NHANES is a nationwide survey conducted annually for the purpose of collecting health and diet information from a representative, non-institutionalized U.S. population. NHANES is unique in that it combines interviews, physical examinations, and laboratory evaluations to obtain a large amount of quantitative and qualitative data. Information on NHANES survey methods are described further in detail elsewhere [38]. Briefly, the survey examines about 5,000 persons each year from various counties across the country. The country is divided into a total of 30 primary sampling units (PSUs), of which 15 are visited each year. The complex survey design assigns a weight to each individual as a function of their probability of being randomly selected into the study and these weightings are taken into account when building our regression models. All participants provided a written informed consent in agreement with the Public Health Service Act prior to any data collection. Household questionnaires, telephone interviews, and examinations conducted by healthcare professionals and trained personnel were utilized to collect data.

### Study participants and exclusion criteria

The 2013–2014 NHANES cycle collected data on 10,175 individuals. We restricted our analysis to adults age 18 and older. We restricted our analysis to adults due to the fact that HTN in the pediatric population is a rare outcome, and would not provide a sufficient sample size for robust analysis. Additionally, pediatric HTN is unlikely to be related to low level, chronic environmental exposures, but rather has been shown to be strongly linked with genetic conditions, and acute, high exposure levels of environmental contaminants [39, 40]. From these remaining individuals, analysis was restricted to men and women with valid blood pressure readings, as well as complete information on demographic, anthropometric, questionnaire, and laboratory variables including BMI, alcohol use, diabetes status, education level, hypercholesterolemia status, insurance coverage status, creatinine and albumin concentrations, race, smoking status, and HTN status, resulting in a final analysis sample size of 935.

### Quantification of TCPy, oxypyrimidine, and para-nitrophenol

Due to the increased cost and technical difficulty in quantifying parent compounds (chlorpyrifos, methyl chlorpyrifos, diazanon, parathion, methyl parathion) in the plasma, the NHANES census collected data on readily available and easier to obtain urinary metabolite concentrations. Studies have shown that these metabolites serve as reliable proxies for parent compounds. TCPy, oxypyrimidine, and *para*-nitrophenol were quantified and extracted from the urine matrix of 935 participants using an automated solid phase extraction system. Selective separation of the analytes was achieved using high-performance liquid chromatography with a gradient elution program. Sensitive detection of the analytes was performed by a triple quadrupole mass spectrometer with a heated electrospray ionization source. Final analyte concentrations were dichotomized to either above the detection limit, a value of 0.15 µg/L, or below the detection limit. A further detailed description on laboratory procedures can be found elsewhere [41].

### Defining demographic variables

Methods for questionnaire data collection are described in the NHANES procedures guide [42]. Participants were classified according to highest level of education attainment, insurance coverage status, smoking status, alcohol use, diabetes status, cholesterol status, and HTN status. Highest level of education attainment was based on responses by participants during the home interview. Insurance status and smoking status were recorded as a yes or no response from the home interview. Alcohol use was defined as a yes for individuals who said they drink at least 2 or more alcoholic drinks a day. Diabetes status was defined as a fasting serum glucose greater than 126, having answered yes to taking diabetic medications, or being told by a physician they have diabetes. Hypertension status was defined by at least 4 separate systolic and/or diastolic blood pressure readings greater than 140 mmHg and/or 90 mmHg respectively, having been told by a doctor one has hypertension, or is currently taking hypertension medications. Cholesterol status was defined by whether or not a person was told he/she has high cholesterol by a physician, or if that person is currently taking hypercholesterolemia medications.

### Statistical analyses

**Fig. 1:**
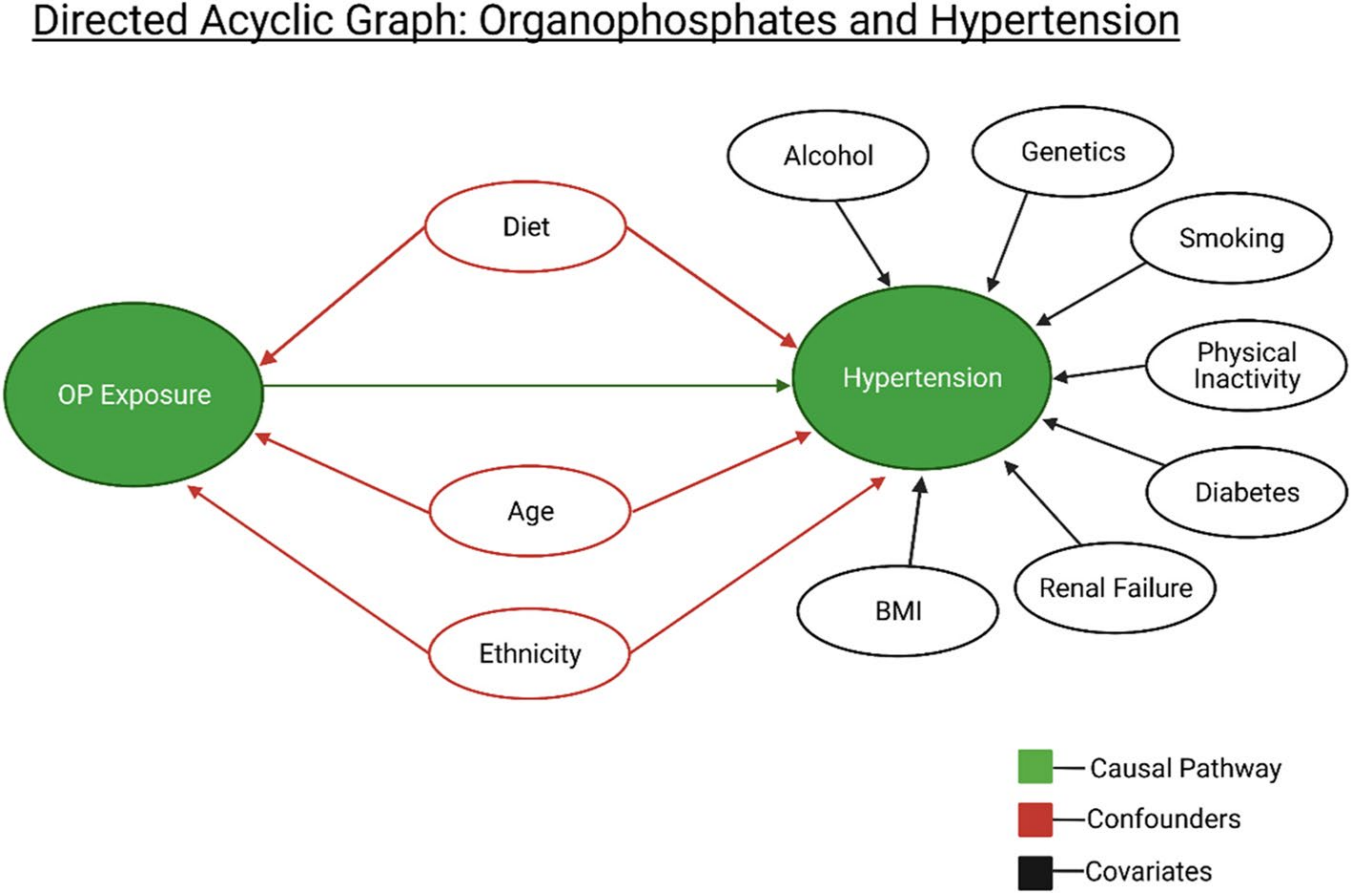
Directed acyclic graph depicting the proposed relationship between OP exposure and HTN. The causal pathway is depicted with the green arrow. Potential founders are depicted along the red arrows. Covariates known to be associated with risk of HTN are along the black arrows

Continuous variables were compared using one-way ANOVA, while categorical variables were compared using the Chi-squared test. Multivariable, ordinary least squares regression models were used to measure the association between the urinary concentrations of OP metabolites and blood pressure. We controlled for potential confounders including race, age, BMI, creatinine levels, diabetes status, education level, smoking status, and hypercholesterolemia based on results from literature searches (Fig. 1). Metabolite values were divided into quartiles for logistic regression analysis, to evaluate the association between quartile levels of each metabolite and the odds of HTN. Oxypyrimidine values below the 75^th^ percentile were detected by the analyzer as 0.70, and therefore we divided oxypyrimidine groups into below the 75^th^ percentile and above the 75^th^ percentile. The lowest quartile was used as the reference in each case, and these results are presented as supplementary information.

All statistical analyses were performed using SAS 9.4 and SUDAAN software packages accounting for the complex survey design of NHANES [43]. A *p*-value < 0.05 was used as the criterion for significance.

## Results

Demographic tables for our cohort are presented in Tables 1, 2, 3, 4 and 5. The mean age for our cohort was 49.3 ± 0.57, and roughly half of individuals were men vs. women. At least 55% of our cohort received some college degree or above. Table 2 shows the demographic breakdown of the cohort stratified by quartile of TCPy exposure. We observed a significant difference among smoking status between quartiles of TCPy. Table 3 shows the demographic breakdown by quartile of *para*-nitrophenol exposure, and Table 4 shows the demographic breakdown by individuals below the 75^th^ percentile of oxypyrimidine exposure vs. those above the 75^th^ percentile. Table 5 shows the demographic breakdown of the cohort by HTN status. We observed significant differences between hypertensive vs. normotensive individuals for age, race, education, hypercholesterolemia status, mean *para*-nitrophenol concentration, and mean values for systolic and diastolic blood pressure. In our regression analysis of the total cohort, we observed a significant, inverse association between TCPy and systolic blood pressure (β-estimate = -0.16, *p* < 0.001) and diastolic blood pressure (β-estimate = -0.15, *p* < 0.001). The interpretation of these estimates would be that for every 1 unit increase in TCPy concentration, we would expect a 0.16 mmHg and 0.15 mmHg decrease on systolic and diastolic blood pressure, respectively. Furthermore, we observed significant interactions between TCPy and ethnicity on systolic blood pressure (β-estimate = 1.46, *p* = 0.0036). This interaction was observed within the Mexican–American race when using Caucasian-Americans as the reference group. The interpretation for this interaction is that when holding all other variables at zero, we expect an increase on systolic blood pressure of 1.46 mmHg when comparing Mexican-Americans exposed to TCPy to Caucasian-Americans. Analysis with *para*-nitrophenol revealed a significant, positive association with systolic blood pressure (β-estimate = 0.03, *p* = 0.02), and an inverse association with diastolic blood pressure (β-estimate = -0.09, *p* < 0.001). A significant interaction was observed between *para-*nitrophenol and BMI on systolic blood pressure (β-estimate = 0.43, *p* = 0.01), and between *para*-nitrophenol and ethnicity on diastolic blood pressure (β-estimate = 2.19, *p* = 0.006). Significant interaction terms were observed between oxypyrimidine and race (β-estimate = -1.73, *p* < 0.001), as well as oxypyrimidine and BMI (β-estimate = 1.51 *p* < 0.001) on systolic blood pressure. We also observed significant interactions between oxypyrimidine and age (β-estimate = 1.96, *p* = 0.02), race (β-estimate = -3.81 *p* = 0.004), and BMI on diastolic blood pressure (β-estimate = 0.72, *p* = 0.02). Lastly, we performed multivariable logistic regression to model the odds of hypertension, at quartile levels of each OP metabolite. The lowest quartile was used as the reference in each case. Results from our logistic regression revealed a significant association between the odds of HTN and TCPy (OR = 0.65, 95% CI [0.43,0.99]) and no significant associations between urinary concentrations of oxypyrimidine, and *para*-nitrophenol (Additional file 1: Supplementary tables 1,2 and3).

**Table 1:**
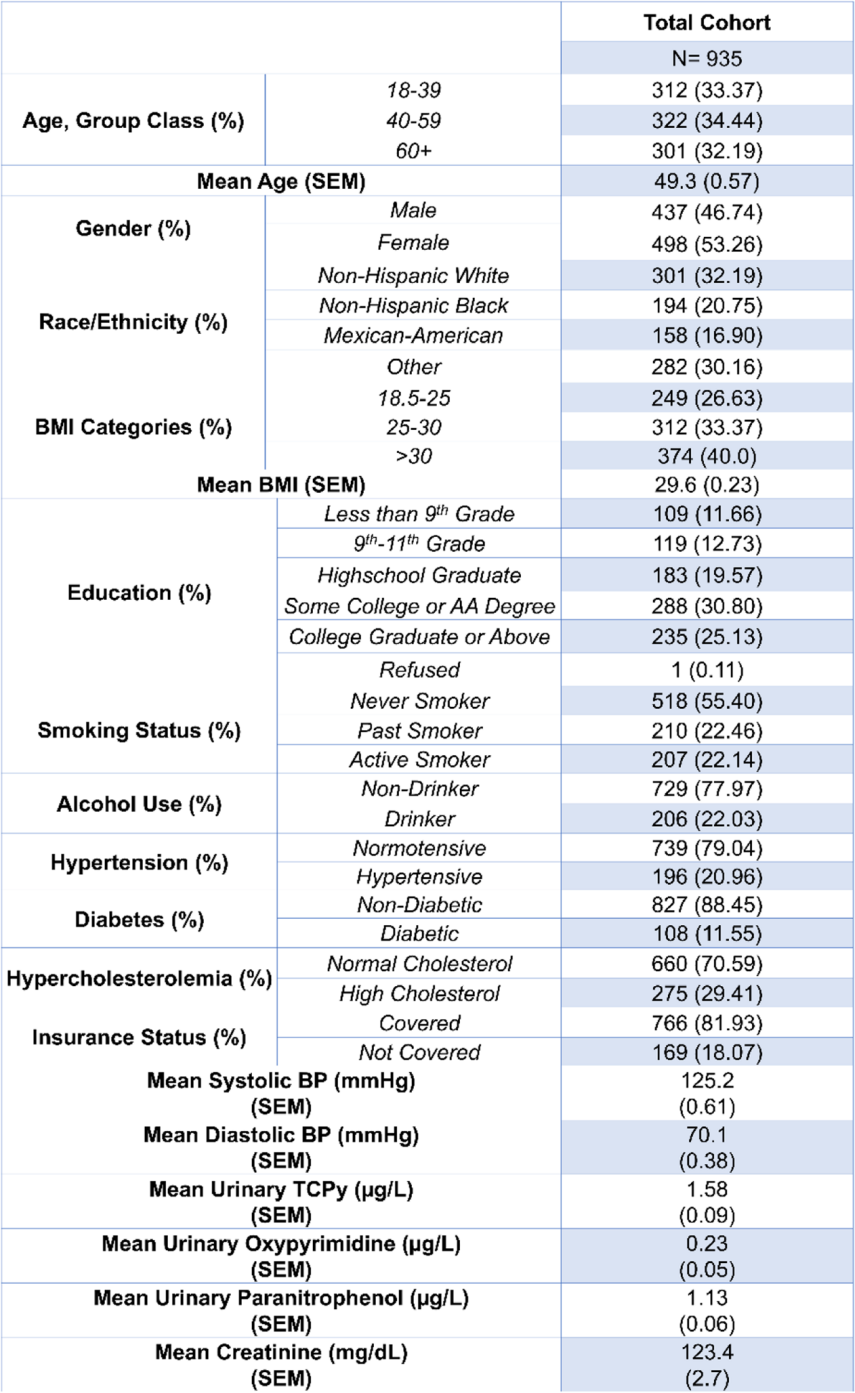
Demographic and Laboratory Data for the Total Cohort

**Table 2:**
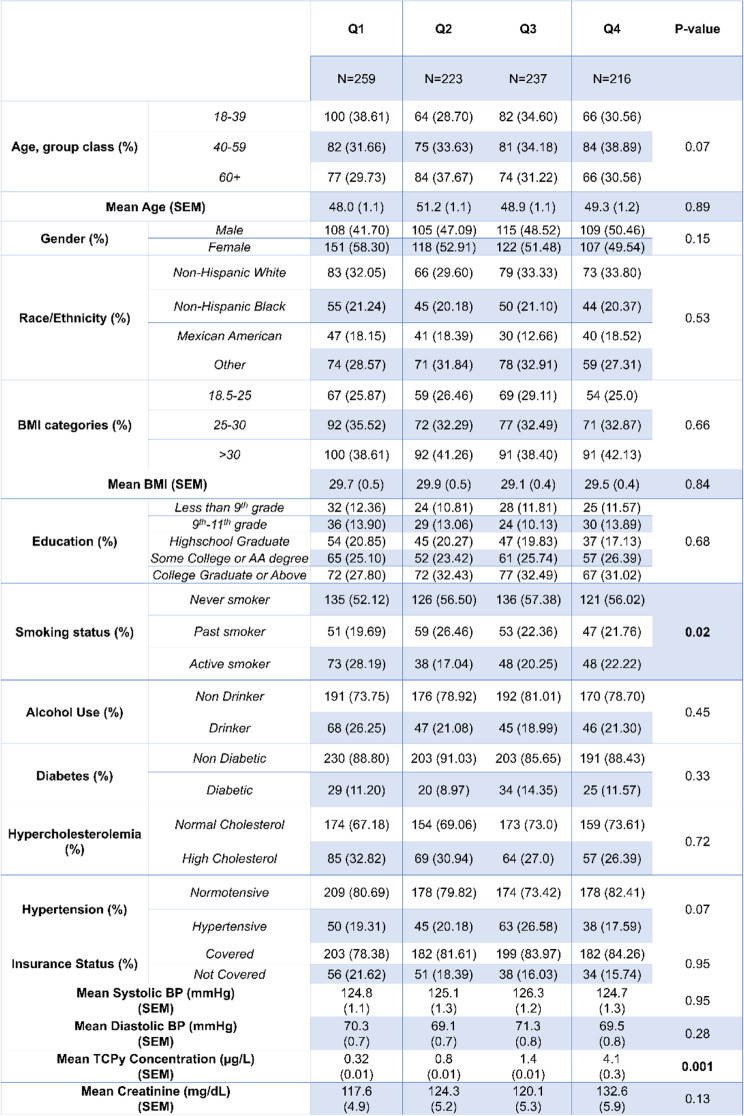
Demographic and Laboratory Data by Quartiles of TCPy

**Table 3:**
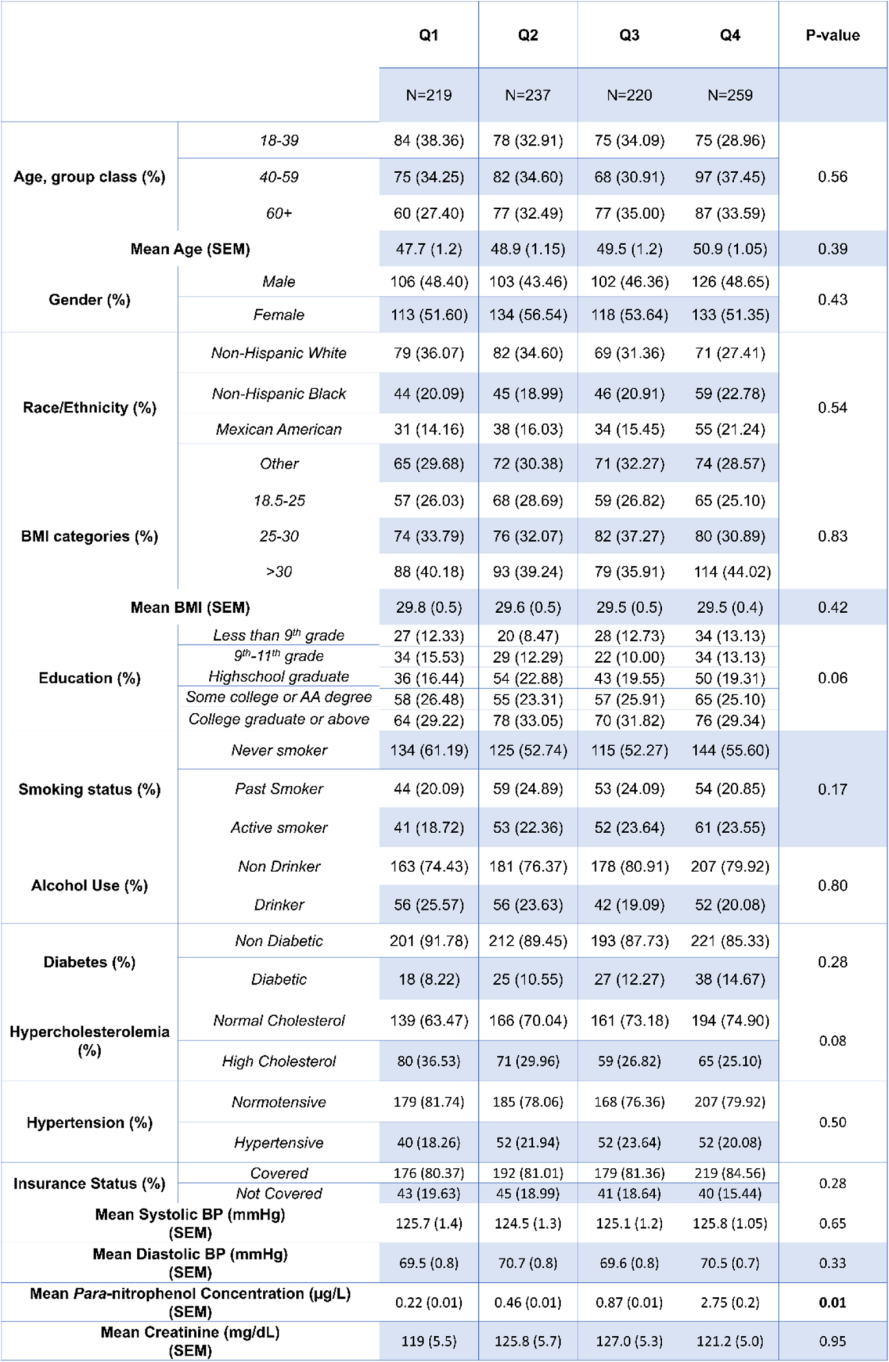
Demographic and Laboratory Data by Quartiles of *Para-*nitrophenol

**Table 4:**
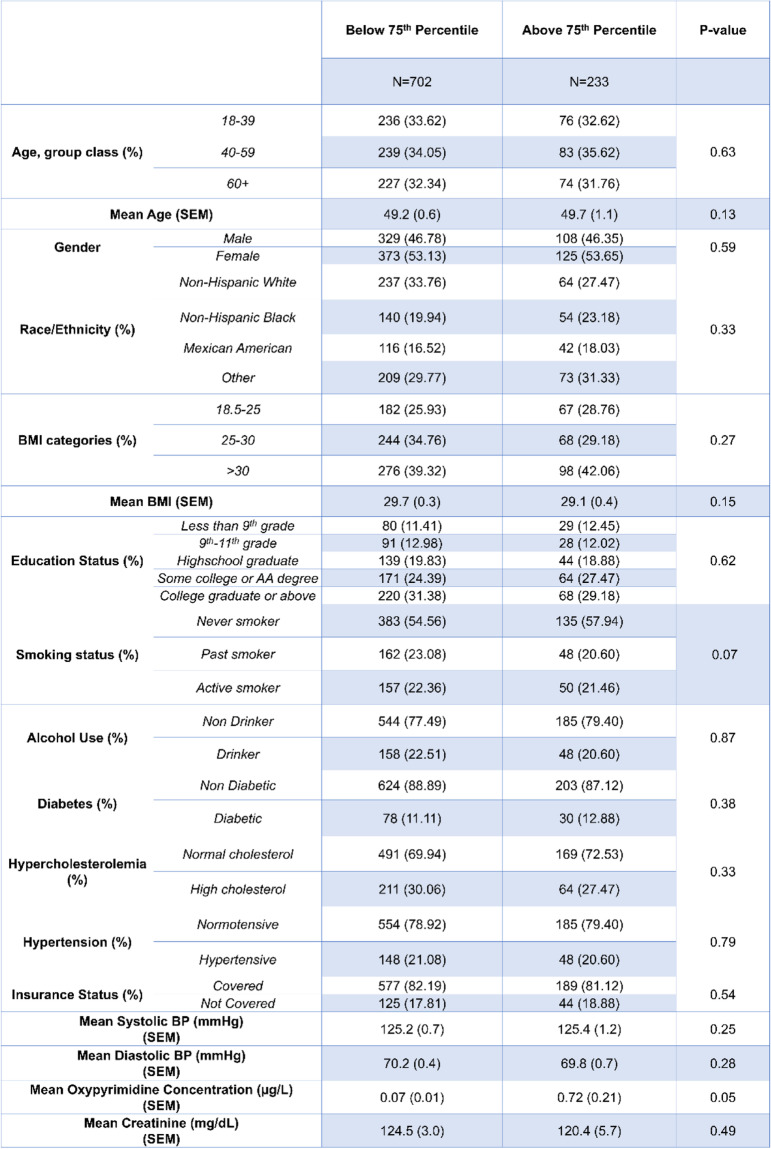
Demographic and Laboratory Data by Oxypyrimidine Percentile

**Table 5:**
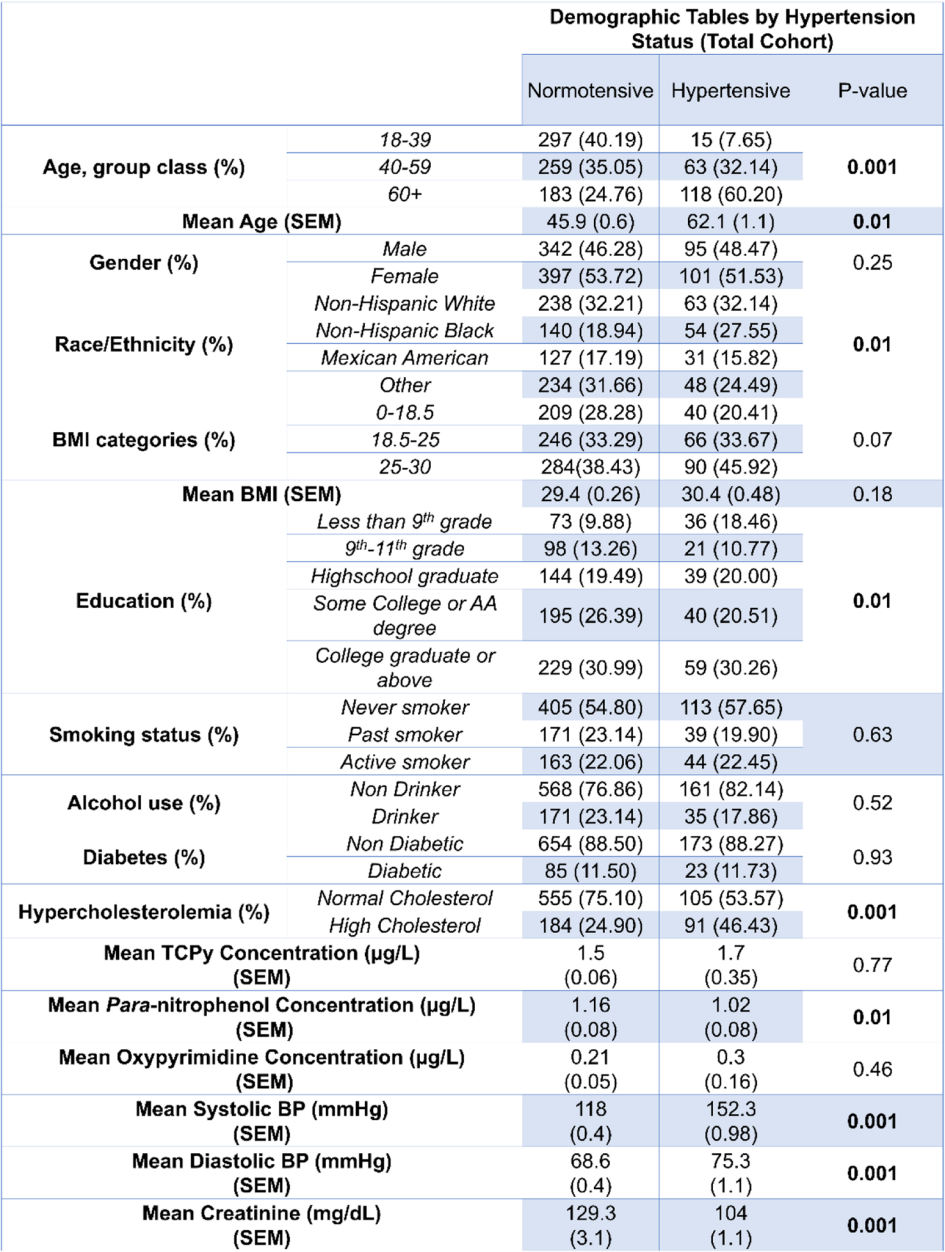
Demographic and Laboratory Data by Hypertension Status

## Discussion

Our preliminary findings support data from previous studies suggesting a link between OP insecticide exposure and blood pressure dysregulation. We observed significant associations between odds of HTN and TCPy, whereas null associations were observed between *para*-nitrophenol and oxypyrimidine with HTN. A potential explanation for these differences in associations among the metabolites may be due to differences in their measured concentrations. For example, TCPy is more readily quantified in the environment compared to oxypyrimidine and *para*-nitrophenol. As a result, our significant association observed between TCPy and HTN may be due to a larger effect size within TCPy analyses compared to the *para*-nitrophenol and oxypyrimidine analyses. Furthermore, it is also possible that the population-level exposures to oxypyrimidine and *para-*nitrophenol are not strong enough to promote an individual into HTN, though they are associated with changes in continuous blood pressure. We additionally observed significant interactions between OP exposure and BMI, age, race on blood pressure. It has been demonstrated that OP insecticides and other environmental chemicals commonly found with OPs (e.g. herbicides, heavy metals, PCBs) can sequester within the biological fat compartment, specifically within adipocytes [44, 45]. In this case, the fat compartment can serve as a reservoir for continued exposure, beyond the initial time of contact. Thus, studies have shown that for varying levels of BMI, the adverse effects of exposure to a chemical can be significantly more pronounced in individuals with higher BMI, because these individuals trap more chemical within their bodies compared to lower BMI individuals, given a same initial exposure of chemical. The interaction between age and OP exposure is possibly due to the fact that older individuals generally have a longer exposure window compared to younger individuals. Additionally, endogenous levels of protective enzymes and metabolic processes wanes with age [46]. Specifically, levels of liver paroxonase enzymes that are responsible for metabolizing OPs wane with increasing age, and therefore older individuals might be more likely to experience adverse health effects of Ops [47, 48]. In particular, the paroxonase-1 is a polymorphic liver and plasma enzyme that catalyzes the breakdown of all three parent OPs in question to their respective metabolites, and differential levels of PON1 in human and animal studies are believed to be important determinants of OP toxicity [49]. Studies have also shown differences in expression levels of parxoxonase enzymes between ethnic groups, and this may in part explain the interaction between ethnicity and OP exposure on blood pressure [50].

There are several potential mechanisms that have been hypothesized explaining the association between OP insecticides and blood pressure dysregulation. It is important to note that acetylcholinesterase inhibition represents only one part of the complete toxicological profile of OP insecticides, which remains to be fully elucidated. To date, there are a limited number of studies that have assessed biological effects of chlorpyrifos, diazinon, and parathion within organ systems regulating blood pressure, and the majority of those studies have investigated chlorpyrifos’ effects. According to the CDC, exposure levels of chlorpyrifos within the general population aren’t expected to significantly inhibit acetylcholinesterase and cause overt cholinergic toxicity [51]. However, there may be subtle biological changes occurring with prolonged, chronic OP pesticide exposure, and effects of OPs at the cellular level within various organs may be related to blood pressure dysregulation. Organophosphates were originally designed as potent neurotoxic agents, and recent in vitro and in vivo animal studies suggest that effects within the central nervous system on neuronal morphogenesis, neurotransmission, and behavior may occur at systemically nontoxic doses or at doses of chlorpyrifos that do not result in readily apparent changes cholinergic pathways [51]. These neuronal pathways (many of which are located in the hypothalamus), rely on the integrity of synapses and neurotransmitter function to regulate the sympathetic nervous system independent of cholinergic pathways, which in turn regulates blood pressure. Most notably, vasopressin, angiotensin II, and leptin hormones act as key effector hormones within the paraventricular nucleus of the hypothalamus [52, 53]. Chlorpyrifos exposure has been shown experimentally to not only increase circulating levels of these hormones, but also bind to their receptors in vitro [54, 55]. These receptors and neurotransmitters belong to pathways that travel from the hypothalamus to the brainstem, which sends outputs to various peripheral organs to regulate blood pressure. Through these actions chlorpyrifos can affect the activity and expression of these pathways, and ultimately affecting blood pressure.

Organophosphate insecticides like chlorpyrifos, diazinon, and parathion have also been shown to affect expression of numerous micro RNAs (miRNAs) in vivo and in vitro [56, 57]. Micro RNAs are short, noncoding RNA molecules that regulate gene expression at the level of transcription. Many of these miRNAs are targets of genes in cardiac tissue, neural tissue, and skeletal tissue that control homeostatic processes including blood pressure regulation [58]. Our lab previously found that differential expression of several miRNAs (miR-20a-5p, miR-4763-5p, and miR-4709-3p) that regulate vascular remodeling, immune pathways, and cardiac function are implicated in the pathogenesis of hypertension [59]. Thus, another possible mechanism through which OP insecticides affects blood pressure is through effects on miRNA-dependent pathways.

Organophosphates have also been shown to induce oxidative stress in various organ systems, a process that may damage to the integrity of these systems and result in aberrations in blood pressure control [60]. Chlorpyrifos, diazinon, and parathion have all experimentally been shown to increase levels of reactive oxygen species in the heart, kidneys, liver, and brain [61–65]. These OPs have also been shown to induce inflammation through upregulation of cytokines and perturbations in the gut microflora, and recent studies have implicated dysregulation of the gut microbiome in HTN pathogenesis [66, 67]. It is important to note that many of these studies were conducted with acute OP exposure levels, and thus future studies using chronic exposure levels and chronic time durations are warranted to assess induction of oxidate stress and inflammation, and what biological effects these processes have on blood pressure.

It is also important to note that TCPy, oxypyrimidine, and *para*-nitrophenol have their own toxicological profiles in various organ systems, independent of acetylcholinesterase inhibition [68, 69]. If the relationship between OP metabolites and blood pressure is due their direct effects (in conjunction with or independent of parent compound effects), then future studies examining the individual effects of these OP metabolites and the parent compounds on blood pressure are warranted. Lastly, chronic chlorpyrifos exposure has been shown to alter brain development and neuronal morphogenesis of developing fetuses in absence of significant acetylcholinesterase inhibition [70, 71]. These in utero exposures may also contribute to the effect of OP insecticides on blood pressure and may even predispose individuals to HTN, and future developmental studies are warranted to test this idea.

The present study has a number of strengths. We incorporated a large number of men and women representative of the general U.S. adult population, and we were able to characterize the association between blood pressure and everyday exposure levels of TCPy, oxypyrimidine, and *para*-nitrophenol. Unlike this study, many previous studies lack generalizability due to the selection of their study populations, which mostly include occupationally exposed pesticide applicators, and agricultural subpopulations living in areas of high OP pesticide concentrations. Additionally, previous studies have relied on using dialkyl phosphates (DAPs) as proxies for OP exposure. Unlike TCPy, oxypyrimidine, and *para*-nitrophenol, DAPs are not unique to any one parent compound, and are a result from metabolism of a number of OP insecticides, making them a less reliable proxy for parent compound exposure. Another strength lies in the oversampling methods of NHANES, which allowed for sufficient sample sizes of minority populations being recruited (Mexican–American, African-American, Asian-American). These groups have been traditionally difficult to include in population-level studies, and when they are included in small numbers there isn’t enough power to estimate main effects with confidence. Through oversampling, we were able to examine main effects of OP exposure on blood pressure within these groups, and also examine interaction effects between OP exposure and race/ethnicity on blood pressure.

The current study has several limitations. Due to the cross-sectional nature of this study, we are unable draw any causal relationships between the exposure to TCPy, oxypyrimidine, *para*-nitrophenol, and blood pressure outcomes. Furthermore, because we are measuring urinary concentrations of metabolites as a proxy for parent compound exposure, we are unable to quantify the true relationship between the parent compounds and blood pressure. The detection frequencies of oxyprimidine and *para*-nitrophenol metabolites are relatively small compared to TCPy, and this may affect the power and precision of our estimates when extrapolating our findings to the general population. Additionally, TCPy, oxypyrimidine, and *para*-nitrophenol are relatively stable in the environment, and thus it is likely that quantified metabolites come not only from direct exposure, but also from a variety of sources such as residues on foods that accumulate overtime. Thus, it is possible that the estimated exposure to the parent compounds is overestimated when using these metabolites as surrogates. Furthermore, dose-response relationships are crucial to understanding the biological effects of insecticide exposure. Both the dose and duration of exposure to insecticides can have varying outcomes on blood pressure, and may also depend on inherent biological and sociodemographic variables such as age, lifestyle practices, and preexisting comorbities [72, 73]. It therefore stands to reason that the concentrations of OPs used in previous toxicological studies may have different effects on blood pressure when compared to concentrations seen at everyday levels. Many of the in vitro and in vivo studies use high doses of insecticides to ensure an effect is measured, but dose–response studies using environmentally representative concentrations are lacking. Additionally, many of these studies measured effects of single OPs on blood pressure. In the general population, individuals are exposed to a wide variety of toxicants daily, and while restrictions and bans have been placed on some insecticides, a multitude of novel insecticides are manufactured yearly with limited toxicological data. The mixture effects of these chemicals in our systems may have antagonist or even synergistic effects on various organs regulating blood pressure, depending on the ratio of chemicals [74]. Future studies including environmentally relevant doses of OPs and inclusion of compound mixtures with various insecticides and pollutants are warranted.

## Conclusion

Our preliminary findings support a potential role for organophosphate insecticide exposure in the pathogenesis of HTN. Results such as these support initiatives to reduce overuse of insecticides, develop safer insecticide alternatives, and to explore alternative avenues for insect control in lieu of insecticides (e.g. bioengineering of insects, crop rotating, etc.). Additionally, improved protocols and safety standards may be beneficial for individuals who use insecticides, as well as farmers and industries whose use of insecticides leads to global exposures at the population level. Future experiments are warranted to elucidate the biological mechanisms responsible for the association between OP insecticides and blood pressure.

## Supplementary Information

**Additional file 1: Table 1.** Logistic Regression Results between TCPy Quartiles and Hypertension. **Table 2.** Logistic Regression Results between *Para*-nitrophenol Quartiles and Hypertension. **Table 3.** Logistic Regression Results between Oxypyrimidine Percentiles and Hypertension.

## Data Availability

A full list of data sets supporting the results in this research article can be found at: https://wwwn.cdc.gov/nchs/nhanes/continuousnhanes/default.aspx?BeginYear=2013.

## References

[1] Ameta K, Gupta A, Kumar S, Sethi R, Kumar D, Mahdi AA (2017). Essential hypertension: a filtered serum based metabolomics study. Sci Rep.

[2] Tzoulaki I, Iliou A, Mikros E, Elliott P (2018). An overview of metabolic phenotyping in blood pressure research. Curr Hypertens Rep.

[3] Falkner B (2010). Hypertension in children and adolescents: epidemiology and natural history. Pediatr Nephrol.

[4] Zhou D, Xi B, Zhao M, Wang L, Veeranki SP (2018). Uncontrolled hypertension increases risk of all-cause and cardiovascular disease mortality in US adults: the NHANES III linked mortality study. Sci Rep.

[5] Oparil S, Acelajado MC, Bakris GL, Berlowitz DR, Cifkova R, Dominiczak AF, Grassi G, Jordan J, Poulter NR, Rodgers A, Whelton PK (2018). Hypertension. Nat Rev Dis Primers.

[6] Egan BM, Stevens-Fabry S (2015). Prehypertension–prevalence, health risks, and management strategies. Nat Rev Cardiol.

[7] Puar THK, Mok Y, Debajyoti R, Khoo J, How CH, Ng AKH (2016). Secondary hypertension in adults. Singapore Med J.

[8] Kuneš J, Zicha J (2009). The interaction of genetic and environmental factors in the etiology of hypertension. Physiol Res.

[9] Perry I, Whincup P, Shaper A (1994). Environmental factors in the development of essential hypertension. Br Med Bull.

[10] Leonel Javeres MN, Habib R, Judith Laure N, Abbas Shah ST, Valis M, Kuca K, Muhammad Nurulain S. Chronic Exposure to Organophosphates Pesticides and Risk of Metabolic Disorder in Cohort from Pakistan and Cameroon. Int J Environ Res Public Health. 2021;18(5). Epub 2021/03/04. 10.3390/ijerph18052310. PubMed PMID: 33652791; PMCID: PMC7967685. 10.3390/ijerph18052310 PMC7967685 33652791

[11] Valenzuela PL, Carrera-Bastos P, Gálvez BG, Ruiz-Hurtado G, Ordovas JM, Ruilope LM, Lucia A (2021). Lifestyle interventions for the prevention and treatment of hypertension. Nat Rev Cardiol.

[12] Yang MH, Kang SY, Lee JA, Kim YS, Sung EJ, Lee K-Y, Kim J-S, Oh HJ, Kang HC, Lee SY (2017). The effect of lifestyle changes on blood pressure control among hypertensive patients. Korean J Fam Med.

[13] Park SH, Lim JE, Park H, Jee SH (2016). Body burden of persistent organic pollutants on hypertension: a meta-analysis. Environ Sci Pollut Res Int.

[14] Arrebola JP, Fernández MF, Martin-Olmedo P, Bonde JP, Martín-Rodriguez JL, Expósito J, Rubio-Domínguez A, Olea N (2015). Historical exposure to persistent organic pollutants and risk of incident hypertension. Environ Res.

[15] Van Ael E, Covaci A, Das K, Lepoint G, Blust R, Bervoets L (2013). Factors influencing the bioaccumulation of persistent organic pollutants in food webs of the scheldt estuary. Environ Sci Technol.

[16] Alavanja MCR (2009). Introduction: pesticides use and exposure extensive worldwide. Rev Environ Health.

[17] Oostingh GJ, Wichmann G, Schmittner M, Lehmann I, Duschl A (2009). The cytotoxic effects of the organophosphates chlorpyrifos and diazinon differ from their immunomodulating effects. J Immunotoxicol.

[18] Rohlman DS, Ismail A, Bonner MR, Abdel Rasoul G, Hendy O, Ortega Dickey L, Wang K, Olson JR (2019). Occupational pesticide exposure and symptoms of attention deficit hyperactivity disorder in adolescent pesticide applicators in Egypt. Neurotoxicology.

[19] Yan D, Zhang Y, Liu L, Yan H (2016). Pesticide exposure and risk of alzheimer's disease: a systematic review and meta-analysis. Sci Rep.

[20] Humans IWGotEoCRt. IARC Monographs on the Evaluation of Carcinogenic Risks to Humans. Some Organophosphate Insecticides and Herbicides. Lyon (FR): International Agency for Research on Cancer© International Agency for Research on Cancer, 2017. For more information contact publications@iarc.fr.; 2017.

[21] Bilal M, Iqbal HMN, Barceló D (2019). Persistence of pesticides-based contaminants in the environment and their effective degradation using laccase-assisted biocatalytic systems. Sci Total Environ.

[22] Damalas CA, Eleftherohorinos IG (2011). Pesticide exposure, safety issues, and risk assessment indicators. Int J Environ Res Public Health.

[23] Lu MX, Jiang WW, Wang JL, Jian Q, Shen Y, Liu XJ, Yu XY (2014). Persistence and dissipation of chlorpyrifos in Brassica chinensis, lettuce, celery, asparagus lettuce, eggplant, and pepper in a greenhouse. PLoS One.

[24] Crane AL, Abdel Rasoul G, Ismail AA, Hendy O, Bonner MR, Lasarev MR, Al-Batanony M, Singleton ST, Khan K, Olson JR, Rohlman DS (2013). Longitudinal assessment of chlorpyrifos exposure and effect biomarkers in adolescent Egyptian agricultural workers. J Expo Sci Environ Epidemiol..

[25] Eaton DL, Daroff RB, Autrup H, Bridges J, Buffler P, Costa LG, Coyle J, McKhann G, Mobley WC, Nadel L, Neubert D, Schulte-Hermann R, Spencer PS (2008). Review of the toxicology of chlorpyrifos with an emphasis on human exposure and neurodevelopment. Crit Rev Toxicol.

[26] Huen K, Bradman A, Harley K, Yousefi P, Boyd Barr D, Eskenazi B, Holland N (2012). Organophosphate pesticide levels in blood and urine of women and newborns living in an agricultural community. Environ Res.

[27] Cunha AF, Felippe ISA, Ferreira-Junior NC, Resstel LBM, Guimarães DAM, Beijamini V, Paton JFR, Sampaio KN (2018). Neuroreflex control of cardiovascular function is impaired after acute poisoning with chlorpyrifos, an organophosphorus insecticide: possible short and long term clinical implications. Toxicology.

[28] Sánchez-Santed F, Colomina MT, Herrero Hernández E (2016). Organophosphate pesticide exposure and neurodegeneration. Cortex.

[29] Kučera M, Hrabovská A (2015). Cholinergic system of the heart. Ceska Slov Farm.

[30] Milutinović S, Murphy D, Japundzić-Zigon N (2006). Central cholinergic modulation of blood pressure short-term variability. Neuropharmacology.

[31] Mancia G, Grassi G (2014). The autonomic nervous system and hypertension. Circ Res..

[32] Erdogan D, Gonul E, Icli A, Yucel H, Arslan A, Akcay S, Ozaydin M (2011). Effects of normal blood pressure, prehypertension, and hypertension on autonomic nervous system function. Intl J Cardiol.

[33] Samsuddin N, Rampal KG, Ismail NH, Abdullah NZ, Nasreen HE (2016). Pesticides exposure and cardiovascular hemodynamic parameters among male workers involved in mosquito control in East Coast of Malaysia. Am J Hypertens.

[34] Suarez-Lopez JR, Amchich F, Murillo J, Denenberg J (2019). Blood pressure after a heightened pesticide spray period among children living in agricultural communities in Ecuador. Environ Res.

[35] Saldana TM, Basso O, Baird DD, Hoppin JA, Weinberg CR, Blair A, Alavanja MC, Sandler DP (2009). Pesticide exposure and hypertensive disorders during pregnancy. Environ Health Perspect.

[36] Ranjbar M, Rotondi MA, Ardern CI, Kuk JL (2015). The influence of urinary concentrations of organophosphate metabolites on the relationship between BMI and cardiometabolic health risk. J Obes.

[37] Alvarez AA. Chlorpyrifos induces hypertension in rats. Journal of Environmental Chemistry and Ecotoxicology. 2011;3(12). doi: 10.5897/jece11.037.

[38] Prevention CfDCa. NHANES Survey Methods and Analytic Guidelines 2020 [cited 2020 October 26]. Available from: https://wwwn.cdc.gov/nchs/nhanes/analyticguidelines.aspx.

[39] Starr MC, Flynn JT (2019). Neonatal hypertension: cases, causes, and clinical approach. Pediatr Nephrol.

[40] Rodd CJ, Sockalosky JJ (1993). Endocrine causes of hypertension in children. Pediatr Clin North Am..

[41] Prevention CfDCa. NHANES 2013–2014 Laboratory Data Overview 2020 [cited 2020 October 26]. Available from: https://wwwn.cdc.gov/nchs/nhanes/ContinuousNhanes/overviewlab.aspx?BeginYear=2013.

[42] Prevention CfDCa. NHANES 2013–2014 Questionnaire Data Overview 2020 [updated August 24 2020]. Available from:https://wwwn.cdc.gov/nchs/nhanes/ContinuousNhanes/OverviewQuex.aspx?BeginYear=2013.

[43] SAS/ACCESS® 9.4 Interface to ADABAS: Reference. Cary: SAS Institute Inc. https://documentation.sas.com/doc/en/pgmsascdc/9.4_3.5/acadbas/titlepage.htm.

[44] Gutgesell RM, Tsakiridis EE, Jamshed S, Steinberg GR, Holloway AC (2020). Impact of pesticide exposure on adipose tissue development and function. Biochem J.

[45] Jackson E, Shoemaker R, Larian N, Cassis L (2017). Adipose tissue as a site of toxin accumulation. Compr Physiol.

[46] Kaur K, Kaur R (2018). Occupational pesticide exposure, impaired DNA repair, and diseases. Indian J Occup Environ Med.

[47] Manthripragada AD, Costello S, Cockburn MG, Bronstein JM, Ritz B (2010). Paraoxonase 1, agricultural organophosphate exposure, and Parkinson disease. Epidemiology.

[48] Vyskočilová E, Szotáková B, Skálová L, Bártíková H, Hlaváčová J, Boušová I (2013). Age-related changes in hepatic activity and expression of detoxification enzymes in male rats. Biomed Res Int.

[49] Costa LG, Giordano G, Cole TB, Marsillach J, Furlong CE (2013). Paraoxonase 1 (PON1) as a genetic determinant of susceptibility to organophosphate toxicity. Toxicology.

[50] Davis KA, Crow JA, Chambers HW, Meek EC, Chambers JE (2009). Racial differences in paraoxonase-1 (PON1): a factor in the health of southerners?. Environ Health Perspect.

[51] Services USDoHH. Biomonitoring Summary 2017 [updated April 7 2017; cited 2021 February 21]. Available from: https://www.cdc.gov/biomonitoring/Chlorpyrifos_BiomonitoringSummary.html.

[52] de Wardener HE (2001). The hypothalamus and hypertension. Physiol Rev.

[53] Khor S, Cai D (2017). Hypothalamic and inflammatory basis of hypertension. Clin Sci.

[54] Katz EJ, Cortes VI, Eldefrawi ME, Eldefrawi AT (1997). Chlorpyrifos, parathion, and their oxons bind to and desensitize a nicotinic acetylcholine receptor: relevance to their toxicities. Toxicol Appl Pharmacol.

[55] Huff RA, Corcoran JJ, Anderson JK, Abou-Donia MB (1994). Chlorpyrifos oxon binds directly to muscarinic receptors and inhibits cAMP accumulation in rat striatum. J Pharmacol Exp Ther.

[56] Yuan H, Yuan M, Tang Y, Wang B, Zhan X. MicroRNA expression profiling in human acute organophosphorus poisoning and functional analysis of dysregulated miRNAs. Afr Health Sci. 2018;18(2):333–42. 10.4314/ahs.v18i2.18. 10.4314/ahs.v18i2.18 PMC6306958 30602960

[57] Ambali S, Abbas S, Shittu M, Dzenda T, Kawu M, Salami S, Ayo J (2009). Effects of gestational exposure to chlorpyrifos on implantation and neonatal mice. J Cell Animal Biology.

[58] Marques FZ, Charchar FJ (2015). microRNAs in Essential Hypertension and Blood Pressure Regulation. Adv Exp Med Biol.

[59] Dluzen DF, Hooten NN, Zhang Y, Kim Y, Glover FE, Tajuddin SM, Jacob KD, Zonderman AB, Evans MK (2016). Racial differences in microRNA and gene expression in hypertensive women. Sci Rep.

[60] Rodrigo R, González J, Paoletto F (2011). The role of oxidative stress in the pathophysiology of hypertension. Hypertens Res.

[61] Janssens L, Stoks R (2017). Chlorpyrifos-induced oxidative damage is reduced under warming and predation risk: explaining antagonistic interactions with a pesticide. Environ Pollut.

[62] Weis GCC, Assmann CE, Mostardeiro VB, Alves AdO, da Rosa JR, Pillat MM, de Andrade CM, Schetinger MRC, Morsch VMM, da Cruz IBM, Costabeber IH. Chlorpyrifos pesticide promotes oxidative stress and increases inflammatory states in BV-2 microglial cells: A role in neuroinflammation. Chemosphere. 2021;278:130417.10.1016/j.chemosphere.2021.130417 10.1016/j.chemosphere.2021.130417 33839396

[63] Alvarez AA, Ramírez-San Juan E, Canizales-Román A (2008). Chlorpyrifos induces oxidative stress in rats. Toxicol Environ Chem.

[64] Shah MD, Iqbal M (2010). Diazinon-induced oxidative stress and renal dysfunction in rats. Food Chem Toxicol.

[65] Edwards FL, Yedjou CG, Tchounwou PB (2013). Involvement of oxidative stress in methyl parathion and parathion-induced toxicity and genotoxicity to human liver carcinoma (HepG2) cells. Environmental toxicology.

[66] Zhang Y, Jia Q, Hu C, Han M, Guo Q, Li S, Bo C, Zhang Y, Qi X, Sai L, Peng C (2021). Effects of chlorpyrifos exposure on liver inflammation and intestinal flora structure in mice. Toxicol Res (Camb).

[67] Jose PA, Raj D (2015). Gut microbiota in hypertension. Curr Opin Nephrol Hypertens.

[68] Meeker JD, Ryan L, Barr DB, Hauser R (2006). Exposure to nonpersistent insecticides and male reproductive hormones. Epidemiology.

[69] Wang L, Liu Z, Zhang J, Wu Y, Sun H (2016). Chlorpyrifos exposure in farmers and urban adults: metabolic characteristic, exposure estimation, and potential effect of oxidative damage. Environ Res.

[70] Mink PJ, Kimmel CA, Li AA (2012). Potential effects of chlorpyrifos on fetal growth outcomes: implications for risk assessment. J Toxicol Environ Health B Crit Rev.

[71] Rauh VA, Garfinkel R, Perera FP, Andrews HF, Hoepner L, Barr DB, Whitehead R, Tang D, Whyatt RW (2006). Impact of prenatal chlorpyrifos exposure on neurodevelopment in the first 3 years of life among inner-city children. Pediatrics..

[72] Reiss R, Neal B, Lamb JC, Juberg DR (2012). Acetylcholinesterase inhibition dose–response modeling for chlorpyrifos and chlorpyrifos-oxon. Regul Toxicol Pharmacol.

[73] Moser VC (2000). Dose-response and time-course of neurobehavioral changes following oral chlorpyrifos in rats of different ages. Neurotoxicol Teratol.

[74] Moser VC, Casey M, Hamm A, Carter WH, Simmons JE, Gennings C (2005). Neurotoxicological and Statistical Analyses of a Mixture of Five Organophosphorus Pesticides Using a Ray Design. Toxicol Sci.

