# Supplementary material for: The association between organophosphate insecticides and blood pressure dysregulation: NHANES 2013–2014
Source: Environ Health. 2022 Aug 8;21:74. doi: 10.1186/s12940-022-00887-3 (PMC9358881; doi:10.1186/s12940-022-00887-3)

Supplementary Tables

Table 1. Logistic Regression Results between TCPy Quartiles and Hypertension


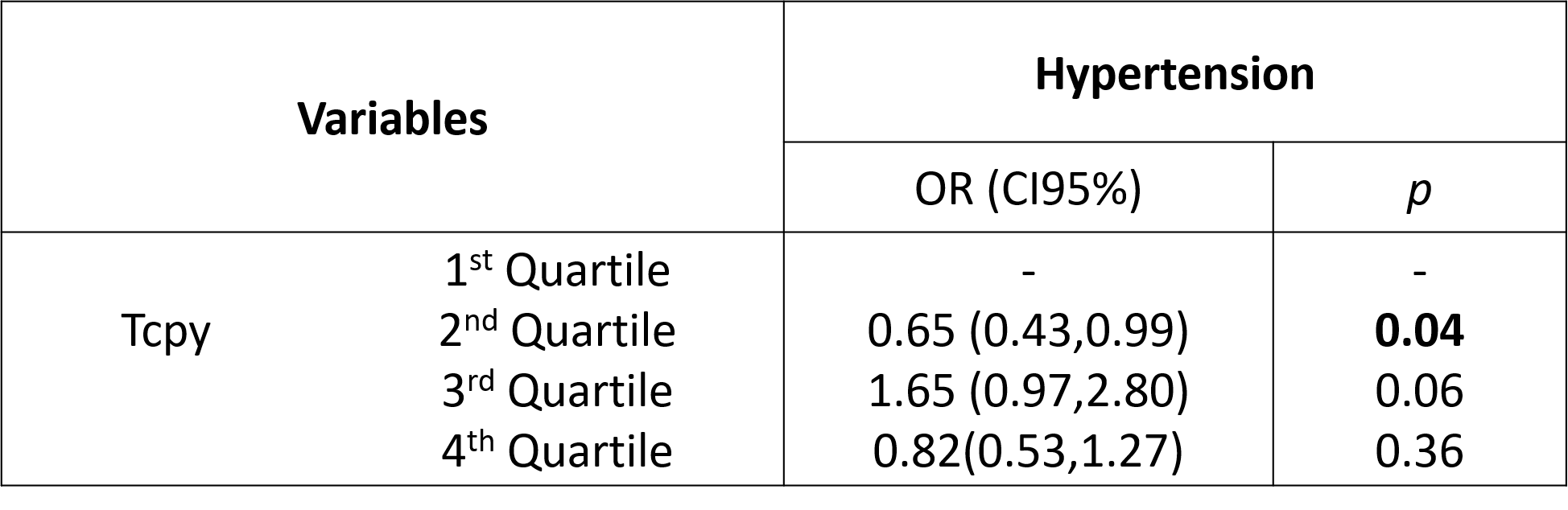


Table 2. Logistic Regression Results between *Para*-nitrophenol Quartiles and Hypertension


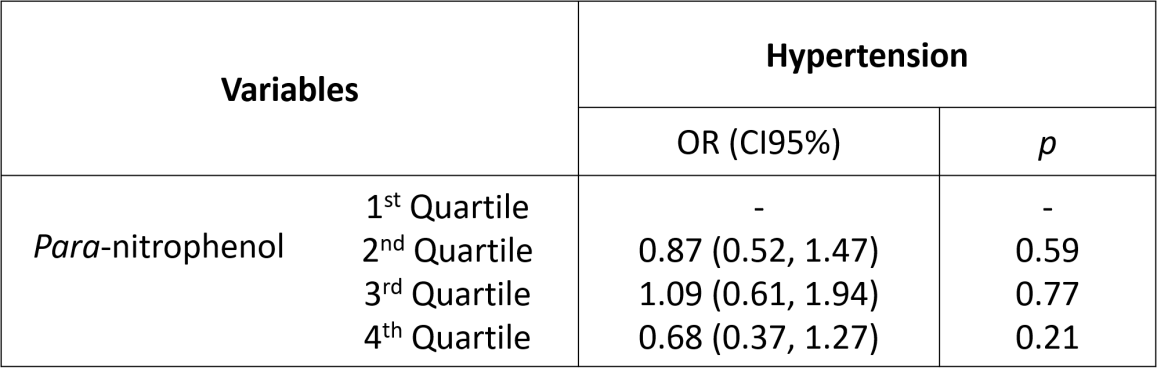


Table 3. Logistic Regression Results between Oxypyrimidine Percentiles and Hypertension
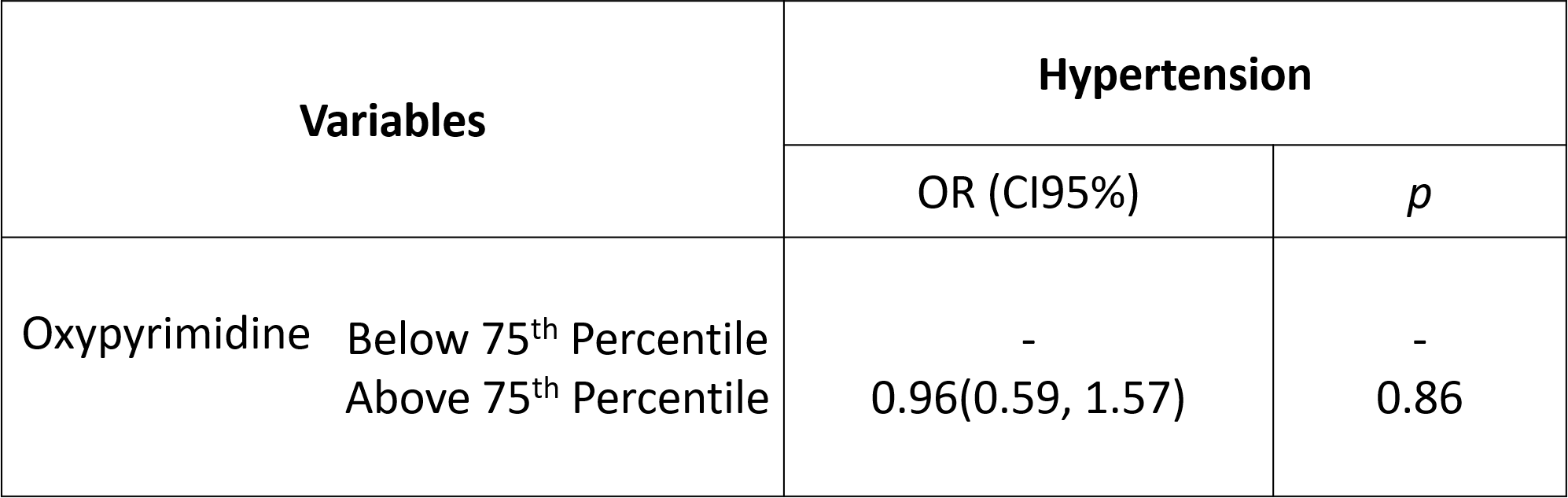

Supplement: Supplementary file 1 — Additional file 1: Table 1. Logistic Regression Results between TCPy Quartiles and Hypertension. Table 2. Logistic Regression Results between Para-nitrophenol Quartiles and Hypertension. Table 3. Logistic Regression Results between Oxypyrimidine Percentiles and Hypertension. [file 12940_2022_887_MOESM1_ESM.docx]
